# Tendon Extracellular Matrix Remodeling and Defective Cell Polarization in the Presence of Collagen VI Mutations

**DOI:** 10.3390/cells9020409

**Published:** 2020-02-11

**Authors:** Manuela Antoniel, Francesco Traina, Luciano Merlini, Davide Andrenacci, Domenico Tigani, Spartaco Santi, Vittoria Cenni, Patrizia Sabatelli, Cesare Faldini, Stefano Squarzoni

**Affiliations:** 1 CNR-Institute of Molecular Genetics “Luigi Luca Cavalli-Sforza”-Unit of Bologna, 40136 Bologna, Italy; manuela.antoniel@gmail.com (M.A.); dandrena@area.bo.cnr.it (D.A.); spartaco.santi@cnr.it (S.S.); vcenni@gmail.com (V.C.); squarzoni@area.bo.cnr.it (S.S.); 2 IRCCS Istituto Ortopedico Rizzoli, 40136 Bologna, Italy; 3 Ortopedia-Traumatologia e Chirurgia Protesica e dei Reimpianti d’Anca e di Ginocchio, Istituto Ortopedico Rizzoli di Bologna, 40136 Bologna, Italy; traina.francesco@gmail.com; 4 Dipartimento di Scienze Biomediche, Odontoiatriche e delle Immagini Morfologiche e Funzionali, Università Degli Studi Di Messina, 98122 Messina, Italy; 5 Department of Biomedical and Neuromotor Sciences, University of Bologna, 40123 Bologna, Italy; mrllcn@unife.it; 6 Department of Orthopedic and Trauma Surgery, Ospedale Maggiore, 40133 Bologna, Italy; domenico.tigani@ausl.bologna.it; 7 1st Orthopaedic and Traumatologic Clinic, IRCCS Istituto Ortopedico Rizzoli, 40136 Bologna, Italy; cesare.faldini@ior.it

**Keywords:** collagen VI, extracellular matrix remodeling, Ullrich congenital muscular dystrophy, Bethlem myopathy, pericellular matrix, cell polarization, NG2 proteoglycan, metalloproteinase 2, cell-extracellular matrix interactions

## Abstract

Mutations in collagen VI genes cause two major clinical myopathies, Bethlem myopathy (BM) and Ullrich congenital muscular dystrophy (UCMD), and the rarer myosclerosis myopathy. In addition to congenital muscle weakness, patients affected by collagen VI-related myopathies show axial and proximal joint contractures, and distal joint hypermobility, which suggest the involvement of tendon function. To gain further insight into the role of collagen VI in human tendon structure and function, we performed ultrastructural, biochemical, and RT-PCR analysis on tendon biopsies and on cell cultures derived from two patients affected with BM and UCMD. In vitro studies revealed striking alterations in the collagen VI network, associated with disruption of the collagen VI-NG2 (Collagen VI-neural/glial antigen 2) axis and defects in cell polarization and migration. The organization of extracellular matrix (ECM) components, as regards collagens I and XII, was also affected, along with an increase in the active form of metalloproteinase 2 (MMP2). In agreement with the in vitro alterations, tendon biopsies from collagen VI-related myopathy patients displayed striking changes in collagen fibril morphology and cell death. These data point to a critical role of collagen VI in tendon matrix organization and cell behavior. The remodeling of the tendon matrix may contribute to the muscle dysfunction observed in BM and UCMD patients.

## 1. Introduction

Ullrich congenital muscular dystrophy (UCMD), Bethlem myopathy (BM), and myosclerosis myopathy (MM) are diseases caused by mutations in each of the three genes (*COL6A1*, *COL6A2*, and *COL6A3*) encoding the extracellular matrix protein collagen VI. Prevalence is estimated at 0.77:100,000 in Bethlem myopathy and 0.13:100,000 in Ullrich CMD [1]. These disorders have a variable clinical severity and a characteristic combination of joint hyperlaxity and muscle contractures. Ullrich congenital muscular dystrophy is a severe disorder characterized by congenital muscle weakness, proximal contractures, and distal laxity; BM is a mild/moderate form characterized by axial and proximal muscle weakness with prominent distal contractures; MM is characterized by slender muscles with firm ‘woody’ consistence and the restriction of movement in many joints [1]. The existence of such a peculiar contracture pattern associated with each clinical form, suggests a role for the collagen VI-based matrix in tendons, which was underestimated until now. 

Tendon architecture comprises few cells, named tenocytes, interspersed within an extracellular matrix (ECM) mainly composed of collagen fibrils organized in longitudinal arrays, whose function is to transmit forces to joint elements without undergoing deformation or damage [2]. Fibrils are made of collagen type I, which represents the main component, and of collagen type III, V, VI, XII, and XIV. Proteoglycan and glycoproteins are also present between fibrils [3]. Tenocytes produce the ECM in tendons and are located along fibrils, lined by a morphological structure known as the pericellular matrix (PCM), a sort of specialized ECM [4,5]. Collagen type V and type VI, fibrillin, and decorin contained in the PCM are reported as regulatory factors for the assembly of collagen fibrils; in addition, also integrins and proteins related to cell adhesion, matrix turn-over, and signal transduction are identified in the PCM [5], which is therefore supposed to drive tendon repair when necessary.

Localization of collagen VI has been determined both at the interfibrillar tendon ECM, appearing as a linking element between coarser collagen type I fibrils, and in the PCM where its fibrils anchor the tenocyte’s membrane and develop extensive networks based on multiple interactions [4,6]. Fibrils appear at the TEM as beaded filaments with a regular 100 nm pace, which are built extracellularly by end-to-end joining of tetramers [6], in turn composed (intracellularly) of two equal sets of three distinct chains forming heterotrimers. There are a variety of these elemental chains; those named alpha1, alpha2, and alpha3 are the most widely expressed and make-up the most common heterotrimer. In the extracellular space, the organization of collagen VI fibrils may vary from extended interconnections to thicker, parallel arrays of beaded fibrils, on the basis of interactions with other ECM elements and cell receptors [6,7,8,9,10]. More recent research led to the identification of additional collagen VI elemental chains in humans, named alpha5 and alpha6, which are similar to alpha3 but are expressed only at specific sites [11,12,13,14]. Regarding tendons, where the most expressed heterotrimer is the alpha1-alpha2-alpha3 [15], alpha5 is found at the myotendinous junction while alpha6 is not expressed.

While we previously demonstrated that collagen VI, through its interaction with the *CSPG4*/NG2 transmembrane proteoglycan, regulates specific cellular functions, including cell polarization and migration, the impact of mutations in collagen VI genes in human tendon function has been poorly explored, mainly due to the difficulty in obtaining biopsies from patients’ tendons [16]. So far, only one study on tendon fibroblasts of a UCMD patient is reported [16]. In this paper we confirm the matrix tendon alterations in another UCMD patient with a mutation in the *COL6A1* gene and show for the first time the presence of a dysfunctional fibrillogenesis and an altered cell behavior in tendon fibroblasts of a patient with BM phenotype. 

In this paper, we demonstrate for the first time that two myopathies, the severe Ullrich and the benign Bethlem, representing opposite ends of the collagen VI-related myopathies spectrum, have similar alterations of the tendon matrix, which also involves, in addition to the collagen VI, collagen I and XII. By in vitro studies we show that alterations in the collagen VI network affect cell polarization and migration.

## 2. Material and Methods

### 2.1. Patients

We obtained a biopsy of the pedidium tendon from a UCMD patient (UCMD) with heterozygous mutation in *COL6A1* [17], and a biopsy of the piriformis tendon was obtained from a BM patient with *COL6A2* exon 6 c.802 G>A het (p.Gly268Ser) (P1 in [18]) that received surgical treatment for a femur fracture. Two piriformis and two pedidium tendons were obtained from healthy volunteers subjected to surgical intervention. Sample processing followed previously described procedures [16]. All subjects gave their informed consent before they participated in the study. The study was conducted in accordance with the Declaration of Helsinki, and the protocol was approved by the Ethics Committee at the Rizzoli Orthopedic Institute (project identification code, PG0006743, approval date: 5th July, 2017).

### 2.2. Tendon Cell Cultures

Tenocytes, the mature cell type of the tendon, have a low proliferative capacity, thus, cultured tendon cells mainly derive from progenitor cells, which display a fibroblast-like phenotype [19]. We used "tendon fibroblasts" to indicate tendon-derived cultures. To obtain tendon fibroblast cultures, tendon fragments were subjected to mechanical dissociation, and maintained in Dulbecco’s modified Eagle medium (DMEM) containing 1% antibiotics plus 10% fetal bovine serum (FBS) [20] and 0.25 mM L-ascorbic acid was added to the medium to allow collagen VI tetramer secretion [21]. Cells were expanded for two passages and stored in liquid nitrogen. All in vitro experiments were performed on cells at passage three to seven. 

### 2.3. Immunofluorescence and Confocal Analysis

Immunofluorescence analysis with anti-collagen VI antibody (Millipore, Temecula, CA, USA) was performed on tendon fibroblast cultures, as previously reported [14]. Cells grown onto coverslips were incubated with antibodies against NG2 proteoglycan, fibronectin (Sigma, Sigma-Aldrich, St Louis, MO, USA), fibronectin (Sigma-Aldrich, St Louis, MO, USA), collagen I (Abcam, Cambridge, UK), collagen XII (Santa Cruz Biotechnology Inc., Santa Cruz, CA, USA), and with FITC (Fluorescein isothiocyanate) or TRITC-(Tetramethylrhodamine) conjugated anti-mouse or anti-rabbit secondary antibodies (DAKO). Cell nuclei were stained with 1 mg/mL DAPI (4′,6-diamidino-2-phenylindole) (Sigma-Aldrich, St Louis, MO, USA). Samples were mounted with an anti-fading reagent (Invitrogen, Carlsbad, CA, USA) and observed with a Nikon epifluorescence microscope. Confocal imaging was performed with a Nikon A1-R confocal laser scanning microscope, equipped with a 60×, 1.4 NA objective and with 405, 488, and 561 nm laser lines to excite DAPI, FITC, and TRITC fluorescence signals. Each final confocal image, of 1024 × 1024 pixels and 4096 gray levels, was obtained by maximum intensity projection of ten optical sections. Co-localization was evaluated on medial optical sections using NIS-Elements software (Nikon, Nikon, Melville, NY, USA). Overlap coefficient *k2*, sensitive to differences in intensity for red signals [22,23], was calculated according to Manders et al. [24]. 

### 2.4. Western Blot Analysis

Cultured tendon fibroblasts were harvested by scraping. The media recovered from the different culture conditions were concentrated with Vivaspin sample concentrators (Vivaspin 2 MWCO10000, GE Healthcare, Amersham, Pittsburgh, PA, USA) according to the manufacturer’s operating procedures. Cell lysates and concentrated culture media were resolved by standard SDS-PAGE, electro-blotted onto a nitrocellulose membrane, and incubated with antibodies against α3(VI), α1(VI), and α5(VI) chains [16]; NG2 proteoglycan antibody (Millipore) was used as previously reported [16]; tenomodulin and actin (Santa Cruz) were used as loading controls. Primary antibodies were followed by incubation with anti-mouse or anti-rabbit horseradish peroxidase (HRP)-conjugated secondary antibodies. Chemiluminescent detection of proteins was carried-out with the enhanced chemiluminescent ECL detection reagent kit (GE Healthcare Amersham, Pittsburgh, PA, USA) according to the supplier’s instructions. 

### 2.5. Gelatin Zymography

To assess gelatinase activity (MMP2 and MMP9), cells were treated with serum-free medium and conditioned media were collected and concentrated (Vivaspin 2, 10.000 MWCO, Sartorius, Göttingen, Deutschland Germany). Gelatinase activity was determined under non-reducing conditions on a 7.5% SDS-polyacrylamide gel containing 2 mg/mL gelatin (Mini-PROTEAN II system; Bio-Rad Laboratories Ltd, Hempstead, UK). Gels were washed in 2.5% Triton X-100 to allow renaturation of MMPs, before they were transferred to a solution containing 50 mM Tris (pH 7.5), 5 mM CaCl_2_, and 1 mM ZnCl_2_, followed by incubation at 37 °C for 18 h. After staining with Coomassie brilliant blue R250 (Bio-Rad Laboratories, Hercules, CA, USA), pro-MMP2 and active MMP2 were observed as white lysis bands produced by gelatin degradation.

### 2.6. Transmission Electron Microscopy Studies

Tendon fragments were fixed with 2.5% glutaraldehyde in 0.1 M cacodylate buffer and 1% osmium tetroxide and embedded in Epon812 epoxy resin following standard procedures. Sections were stained with uranyl acetate and lead citrate and observed with a Jeol Jem-1011 transmission electron microscope operated at 100 kV. For rotary shadowing, tendon fibroblasts were grown onto coverslips and, after confluence, were treated for 24 h with 0.25 mM L-ascorbic acid. In vitro immunolabeling was performed with a polyclonal antibody against α3(VI) chain. Rotary shadowing of immuno-gold labeled samples was performed following reported procedures [25]. Replicas were washed with distilled water, collected on copper grids, and examined with a Jeol Jem-1011 transmission electron microscope operated at 100 kV. 

### 2.7. Quantitative RT-PCR

Total RNA was extracted with TRI Reagent solution (Invitrogen) and then treated with TURBO DNase (Invitrogen, Carlsbad, CA, USA). cDNAs were synthesized using the High-Capacity RNA-to-cDNA Kit (Applied Biosystems, Foster City, CA, USA), according to the manufacture’s protocol. Gene expression was determined by qPCR, using Power SYBR Green PCR master mix (Applied Biosystems). Expression analysis was performed using the Applied Biosystem 7900HT real-time PCR system. The following primers were used: GAPDH, 5’-TCGGAGTCAACGGATTTGGT-3’ (forward) and 5’-TTGCCATGGGTGGAATCATA-3’ (reverse); MMP2, 5’-TGATGGAGAGGCAGACATCA-3’ (forward) and 5’-TACCGTCAAAGGGGTATCCA -3’ (reverse); CSPG4, 5’-GAAGGAGGACGGACCTCAAG-3’ (forward) and 5’-GCTGCTCTTCCACCATTCTC-3’ (reverse). To normalize transcript levels, the internal standard gene *GAPDH* was used, and the ΔΔCt method was adopted to quantify the fold change of expression levels. Each experiment was performed using *n* = 2 independent biological samples from the same patient.

### 2.8. Scratch Wound Healing Assay

Normal, BM, and UCMD tendon fibroblasts were seeded onto coverslips and cultured to confluence in 10% FBS-containing medium for 24 h. A straight scratch simulating a wound was made across the center of the cell monolayer, using a sterile 200-μL pipette tip. After 6 h, cells were fixed with cold methanol and processed for collagen VI and NG2 or Golgin-97 immunofluorescence analysis. For tracking analysis, cells were grown to confluence on tissue culture dishes, and after scratching, phase contrast images were acquired for 20 h at regular intervals of 15 min. Single cell migration was monitored by evaluating the accumulated distance (path length from start to end point) and the Euclidean distance (the shortest distance between start and end point).

### 2.9. Statistical Analysis

Statistical analysis was performed by a Student’s t-test with GraphPad Prism version 8.0.0 for Windows (GraphPad Software, San Diego, CA, USA). The results were considered statistically significant for *p*-values less than 0.05.

## 3. Results

### 3.1. Collagen VI Expression in Tendon Fibroblast Cultures of BM and UCMD Patients

**Figure 1:**
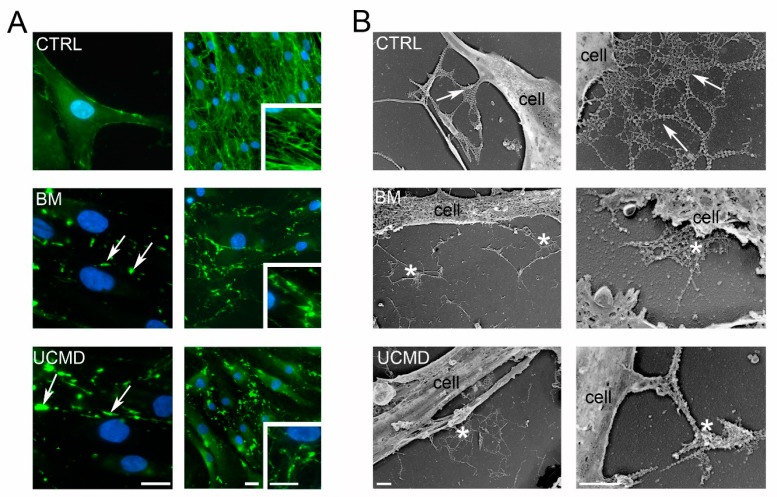
Collagen VI expression in tendon primary cultures of Bethlem myopathy (BM) and Ullrich congenital muscular dystrophy (UCMD) patients. (**A**) Immunofluorescence analysis of collagen VI (green) in proliferating (left panels) and confluent (right panels) tendon cultured fibroblasts from control (CTRL), BM, and UCMD patients. A clear cell membrane association of collagen VI is detectable in normal proliferating cells, while confluent cultures display a filamentous arrangement (inset in right panel). Note the presence of collagen VI aggregates (arrows) both in proliferating and confluent cultures of BM and UCMD cultures. Nuclear staining, DAPI. Scale bar, 10 μm. (**B**) Transmission electron microscopy visualization of rotary shadowed replicas from controls (CTRL; upper panels), BM (middle panels), and UCMD (right panels) showing well extended webs (arrows) associated with the cell membrane in normal control; disorganized webs (asterisks) are evident in BM and UCMD samples. Scale bar, 1 μm.

Collagen VI is a component of the PCM of normal tendon fibroblasts [25]. In order to define whether BM and UCMD patient cells are able to organize a collagen VI pericellular matrix, proliferating tendon fibroblasts from patients and controls were grown in the presence of L-ascorbic acid. Secreted collagen VI was studied by immunofluorescence and Western blot analysis. In normal tendon cultures, collagen VI microfilaments distributed along the tendon fibroblasts processes, while in BM and UCMD cultures, aggregates of collagen VI appeared to mainly deposit among the cells (Figure 1A, left panels). In long-term patient cultures, collagen VI organization displayed a spot-like appearance with respect to the filamentous arrangement of the control (Figure 1A, right panels). The organization of collagen VI was further explored by electron microscopy analysis of rotary shadowed replicas of proliferating cells, immunolabeled with an anti-α3(VI) chain specific primary antibody and a 5-nm colloidal gold-conjugated secondary antibody. In control replicas, typical webs of collagen VI appeared well laid out and anchored to the cell surface along the cell processes, a pattern consistent with the proposed function of collagen VI in mediating the attachment of cells to the substrate. In contrast, UCMD and BM cultures displayed tangled webs and short single microfilaments of collagen VI deposited on the substrate. Aspects of collagen VI microfilaments and web association with the cell processes were rare (Figure 1B).

Western blot analysis of cell lysates and conditioned media from BM, UCMD, and control, in the presence of ascorbic acid, showed comparable amounts of collagen VI α1(VI) and α3(VI) chains (Figure S1A). Changes in expression of the α5(VI) chain were detected in the UCMD cell lysate and medium, with decreased protein level in the cell lysate and an increase in the medium. In BM cells and medium, the α5(VI) chain was comparable with that of the normal control (Figure S1A). 

### 3.2. Collagen VI-NG2 Axis is Disrupted in Tendon Fibroblasts from BM and UCMD Patients

**Figure 2:**
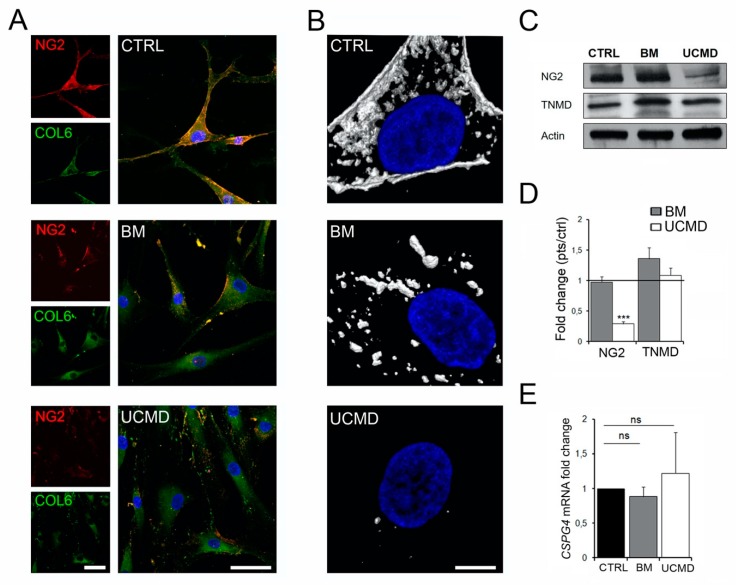
Collagen VI-NG2 axis is disrupted in tendon fibroblasts from BM and UCMD patients. (**A**) Confocal microscopy analysis of tendon fibroblasts double-labeled with anti-collagen VI (COL6, green fluorescence) and NG2 (NG2, red fluorescence) on proliferating samples from control (upper panels), BM (middle panels), and UCMD (lower panels). Collagen VI co-localizes with NG2 at the cell membrane of control cells. Note the moderate reduction of NG2 in BM cells, which correlates with the altered collagen VI signal. NG2 signal is reduced in UCMD cells, while collagen VI staining is dispersed among the cells. Nuclear staining, DAPI. Scale bar, 40 μm. (**B**) Co-localization analysis of the representative perinuclear area in collagen VI-NG2 double-labeled samples, from control (upper panel), BM (middle panel), and UCMD (right panel). White signal identifies areas of high co-localization. Nuclear staining, DAPI. Scale bar, 10 μm. (**C**) Western blot analysis of NG2 in cell cultures of control (CTRL), BM, and UCMD cell lysates shows reduced NG2 expression in the UCMD cells. In BM cells, NG2 is expressed at levels similar to that of the control cell lysate. Tenomodulin (TNMD), used as a marker of tendon cells, and actin, used as a loading control, do not show differences between control and patient cell lysates. (**D**) Densitometric analysis of NG2 and TNMD. Protein levels were calculated as relative intensity with respect to actin. The respective protein levels in BM and UCMD cells were compared to the control (CTRL; showed by the dark line, set as 1) ± SE. *** *p* < 0.001. (**E**) *CSPG4* mRNA analysis in UCMD and BM cells showing no obvious differences with respect to control (CTRL) cells. Data represent mean ± SE of three independent experiments; ns, non-significant.

Given the critical role of the NG2 proteoglycan in mediating the attachment of collagen VI microfibrils to the cells [25], we studied the expression pattern of NG2 in BM and UCMD tendon cultures. Confocal microscopy with anti-NG2 and anti-collagen VI antibodies showed that in control cells NG2 was present at the cell membrane and clearly co-localized with collagen VI. In BM cultures, NG2 proteoglycan showed a clustered distribution that correlated with the anomalous aggregates of collagen VI. In UCMD tendon cell cultures, NG2 staining was barely detectable while collagen VI was apparently not associated with the cell surface (Figure 2A). In agreement with a reduced association of collagen VI with NG2 proteoglycan (Figure 2B), the *k2* co-localization coefficient was reduced in BM and UCMD cells when compared with normal cells (Figure S1B). A consistent reduction of NG2 was also demonstrated by Western blot analysis in UCMD cells, while in BM cells the protein level was similar to that of normal cells (Figure 2C), as indicated by densitometric analysis (Figure 2D). Interestingly, in UCMD, the *CSPG4* mRNA transcript, encoding for NG2 proteoglycan, was comparable with that of the control (Figure 2E), suggesting that the reduced protein amount could be due to a post-transcriptional regulatory mechanism. These data indicate that the organization of endogenous NG2 by tendon cells is affected by the presence of collagen VI mutations, thus potentially having a role in the function of the collagen VI-based pericellular matrix.

### 3.3. Disruption of Collagen VI-NG2 Axis Affects BM and UCMD Cell Polarization During Migration

**Figure 3:**
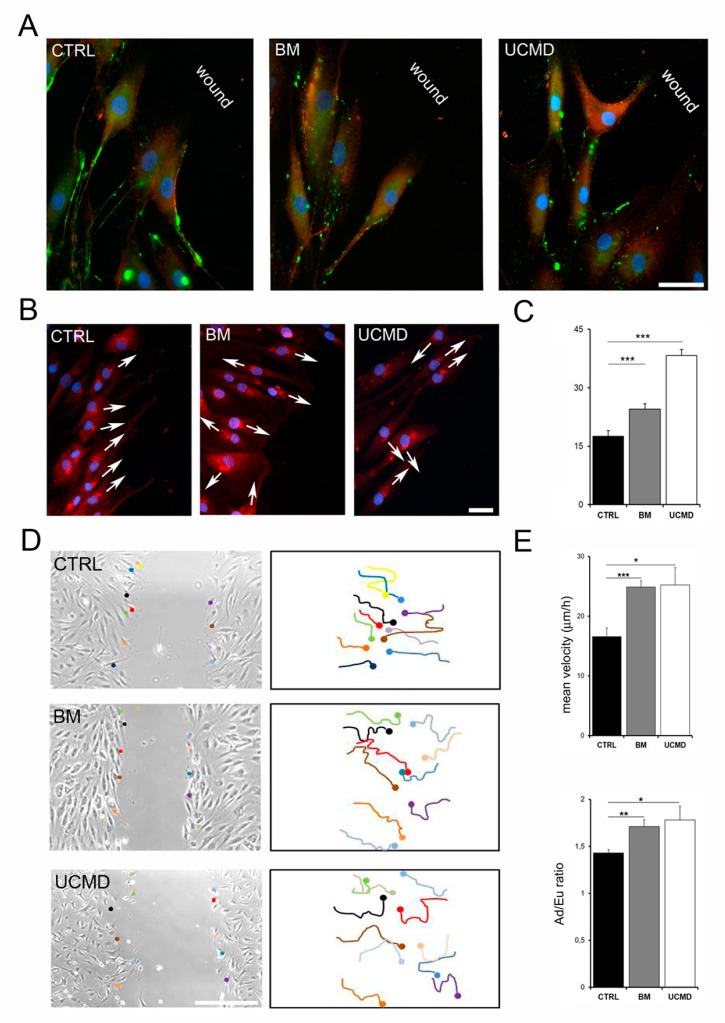
Disruption of collagen VI-NG2 axis affects BM and UCMD cell polarization during migration. (**A**) Immunofluorescence microscopy of collagen VI (COL6) and NG2 double-labeled cells subjected to scratch wound assay. Collagen VI microfilaments and NG2 co-localize at the rear of control (CTRL) cells. In BM and UCMD migrating cells, collagen VI and NG2 staining appears reduced, with a spot-like pattern. Nuclear staining, DAPI. Scale bar, 50 μm. (**B**) Immunofluorescence microscopy of Golgin-97, a marker of the Golgi apparatus, in tendon fibroblasts subjected to the scratch wound assay. In migrating cells of the control (CTRL), the Golgi apparatus was located between the cell’s leading edge and the nucleus. In BM and UCMD samples, cells at the wound edge appear incorrectly oriented (arrows indicate the direction of the single cells). Scale bar, 20 μm. (**C**). The graph indicates the percentage of cells whose Golgi apparatus is not facing the wound. Data represent mean ± SE of three independent experiments. *** *p* < 0.0001. (**D**) Tracking of the individual cell movement during In vitro scratch wound assay. On the left, phase contrast images at T0 of scratched samples. Colored dots identify the position of the nucleus of monitored cells. On the right, graphical representation of the tracks of each cell monitored for 20 h (T20). Scale bar, 0.5 mm. (**E**) Graphs showing the mean velocity (upper) and the ratio of the accumulated distance (Ad) to the Euclidean distance (Eu) (lower) of control (CTRL), BM, and UCMD patient cells. Data represent mean ± SE of three independent experiments. * *p* < 0.05, ** *p* < 0.001.

Given the role of the collagen VI-NG2 axis in stabilizing the lagging end of cells during migration [16], we subjected BM and UCMD tendon fibroblasts to scratch wound assay, to study cell motility and migration in response to in vitro injury. Confluent tendon fibroblast cultures were scratched and after six hours were studied by immunofluorescence analysis. In agreement with previous studies [25], control cells displayed collagen VI at the trailing edge of the moving cells, with typical collagen VI microfilaments anchoring the cell rear to the substrate (Figure 3A). By contrast, BM and UCMD cells showed collagen VI mainly deposited among the cells. Few small aggregates were associated with the cell rear, suggesting that in patient-derived cells the collagen VI-NG2 binding is lost during migration (Figure 3A). To explore its functional consequences, we studied cell polarization using the scratch wound assay. Tendon fibroblasts facing the wound promptly migrated toward the empty space created by the scratch. As a marker of cell polarization, we used Golgin-97, a protein of the Golgi apparatus, which in migrating cells is oriented toward the leading edge with respect to the cell nucleus. As expected, most of the migrating control cells showed uniform orientation towards the wound edge. Strikingly, UCMD cultures displayed a strong increase of incorrectly polarized cells. Although less marked, the number of incorrectly oriented cells was also significant in the BM culture (Figure 3B,C). We monitored the migration of single cells after scratch wounding and imaged cells at constant intervals (15 min) for 20 h. The migration speed of cells facing the wound was assessed by evaluating the ratio between accumulated distance (the sum of the trajectory distances between start and end points) and time. The persistence of directional migration was determined by calculating the ratio of the accumulated distance to the Euclidean distance (the shortest distance between start and end points). Interestingly, both measurements were significantly increased in BM and UCMD patient cells, indicating that, although faster, collagen VI-deficient cells display a random trajectory compared to normal cells (Figure 3D,E). This new experiment adds important information about the mechanism regulated by collagen VI during cell migration. By anchoring the cell rear to the substrate, collagen VI contributes to the stability of cell direction. 

### 3.4. Collagen VI Alterations Affect the ECM Organization in BM and UCMD Tendon Fibroblasts

**Figure 4:**
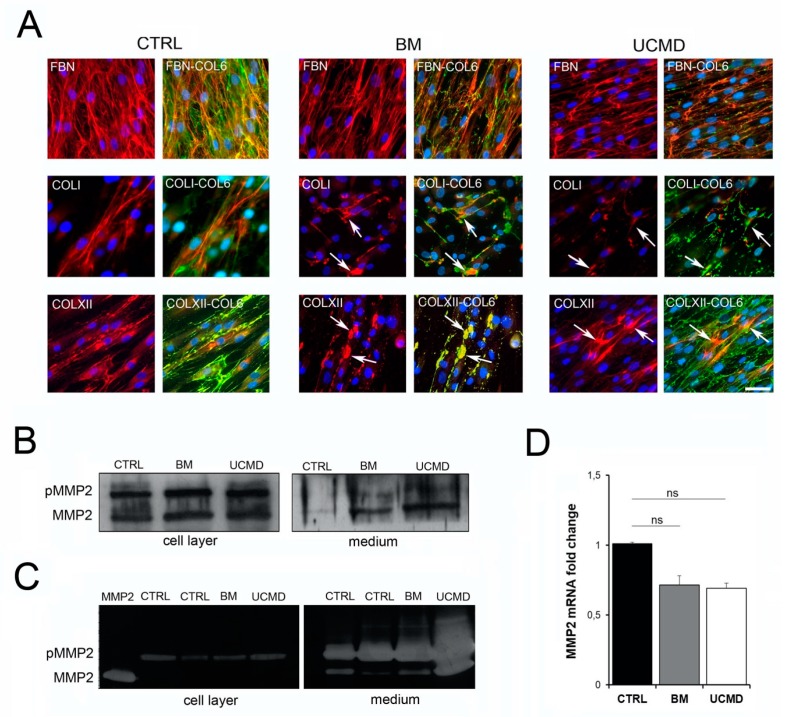
(**A**) Immunofluorescence microscopy of fibronectin (FBN), collagen type I (COLI), collagen type XII (COLXII), and their merge with collagen VI (COL6, green) in control (CTRL), BM, and UCMD long-term cultures. Note the longitudinal pattern of fibronectin fibrils in BM and UCMD samples, when compared with the intertwined pattern in normal cells. In both BM and UCMD cultures, collagen I and collagen XII staining, and their merge with collagen VI, show the presence of protein aggregates (arrows). Nuclear staining, DAPI. Scale bar, 50 μm. (**B**) Western blot analysis of active MMP2 (MMP2) and pro-MMP2 (p-MMP2) in control (CTRL), BM, and UCMD cell layer and medium showing an increase of the MMP2 active form in the conditioned medium of both patient cultures. (**C**) Gelatin zymography of cell layer and conditioned medium of two controls (CTRL), BM, and UCMD patients. MMP2 was loaded at a concentration of 50 mg/mL as a reference to the mobility of the active form. Note the increase of both pro-MMP2 (p-MMP2) and MMP2 activity in the UCMD medium. No obvious differences are detectable in BM cell layer and medium when compared with two controls (CTRL). (**D**) MMP2 mRNA analysis in UCMD and BM cells showing no significant (ns) differences with respect to control (CTRL) cells. Data represent mean ± SE of three independent experiments.

To better define the influence of collagen VI mutations on the assembly of the extracellular matrix, we studied the expression of some collagen VI-related ECM proteins potentially relevant for tendon matrix function. Fibronectin is associated to the pericellular matrix of tenocytes in vivo, and is expressed by tendon fibroblasts in vitro [16]. In normal tendon cultures, a fine network of intertwined fibronectin fibrils was detected, which co-localized with collagen VI fibrils. Similarly, collagen I and collagen XII, two major components of the matrix tendon, displayed a filamentous arrangement that is partially co-distributed with the collagen VI network (Figure 4A, upper panels). In contrast, in BM and UCMD cultures, fibronectin displayed a parallel arrangement, with fibrils running parallel to the long axis of the cells. In addition, the organization of collagen I and collagen XII were affected, as indicated by the presence of aggregates matching with collagen VI anomalous deposits (Figure 4A, middle and lower panels). Furthermore, we investigated the expression and activity of the gelatinase MMP2, which is involved in tendon matrix turnover and remodeling [26]. Western blot analysis showed an increase in expression of the 63 kDa active form of MMP2 in conditioned medium from BM and UCMD patient cultures, while pro-MMP2 levels were unchanged as compared to controls (Figure 4B). In contrast, the expression of MMP2 and pro-MMP2, in cell lysates of the same BM and UCMD cultures, was comparable with normal controls. By gelatin zymography, we found increased MMP2 activity in the conditioned medium of UCMD patient cells, while, in patient cell lysate, it was comparable to that of normal controls. In contrast, BM cell lysate and medium did not show significant changes in gelatinolytic activity, compared to control cells (Figure 4C). The level of MMP2 mRNA transcripts in cells derived from patients was similar to that of normal cells (Figure 4D). These data indicate that in the presence of mutated collagen VI chains, MMP2 may undergo proteolytic activation after secretion in the extracellular matrix, though its activity is differently regulated in cells of BM with respect to that of UCMD patients.

### 3.5. UCMD and BM Tendon Biopsies Show Changes Consistent with ECM Remodeling

**Figure 5:**
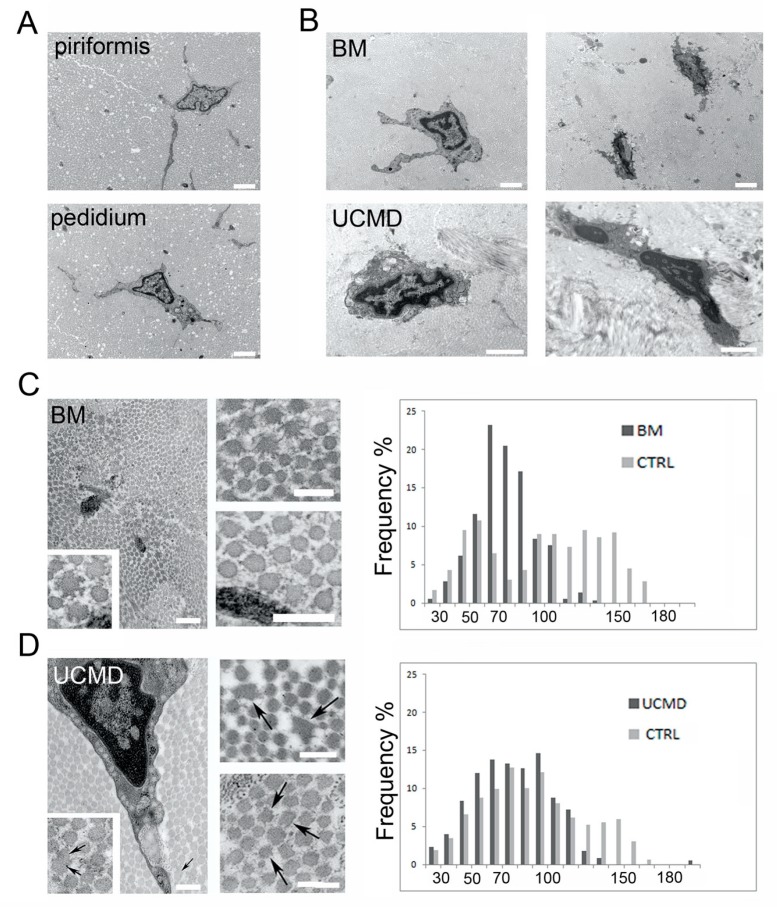
Transmission electron microscopy analysis of tendon biopsies. (**A**) Ultrastructural analysis of normal piriformis and pedidium tendon showing long cellular processes. Scale bar, 1 µm. (**B**) BM and UCMD tenocytes displaying reduced and irregular cellular processes. Necrotic cell with hypercondensed heterochromatin and vacuoles is shown (upper and lower lane, right panels). Scale bar, 1 µm. (**C**) Transmission electron microscopy of cross-sectioned BM piriformis tendon showing altered fibrils with irregular profiles and a ragged appearance (inset). Scale bar, 1 µm. 60 nm. (**D**) UCMD pedidium tendon with altered fibrils (arrows) showing irregular profile. Scale bar, 1 µm. 60 nm.

To gain a better understanding of the tendon matrix in vivo, we performed an ultrastructural analysis on a pedidium tendon biopsy obtained from a UCMD patient and on a fragment of the piriformis tendon obtained from a BM patient and compared the morphology with biopsies obtained from identical tendons of healthy subjects. Normal piriformis and pedidium tendons showed quite similar morphological features, consisting of scattered tenocytes with long cellular processes and well packed collagen fibrils oriented parallel to the major axis of the tendon (Figure 5A). The tendon matrix of both normal tendons was mainly constituted by collagen fibrils of different diameters and few elastin-oxytalan fibers (Figure S1D). The analysis of collagen fibril diameter revealed a bimodal distribution, with fibrils ranging between 20 and 170 nm, and a shift toward large fibers in pedidium (Figure 5C,D). The analysis of the UCMD and BM tendon biopsies showed tenocytes with reduced cell processes and hypercondensed heterochromatin, features of dying cells (Figure 5B). In addition, cross-sectioned BM and UCMD tendon biopsies displayed alterations of the collagen fibril morphology which showed irregular profiles. Groups of "ragged" fibrils were detected in BM (Figure 5C), while scattered large "cauliflower-like" fibrils were often detected in the tendon of the UCMD patient (Figure 5D). These alterations were present both in proximity to the tendon fibroblasts as well as dispersed in the extracellular matrix. The diameter analysis of patients’ fibrils revealed minimal changes to the median values when compared with that of the respective healthy tendon (Figure S1D); however, the frequency of patient fibril distribution was clearly shifted toward smaller diameters (50–100 nm in BM tendon, and 50–120 nm in UCMD tendon), with loss of fibrils having a diameter >140 nm (Figure 5D). 

## 4. Discussion

Patients with collagen VI-related myopathies develop contractures and joint hyperlaxity, which have a significant impact on the patient’s quality-of-life. Identifying the molecular cause(s) of contractures will help in planning therapeutic strategies in collagen VI-related myopathies. Emerging theories point to a tendon dysfunction as the major cause for the onset of contractures [16,27,28]. We had the opportunity to obtain tendon tissue fragments from a BM patient and UCMD patient who received surgical treatment. We found extracellular matrix defects in the tendon biopsy and derived fibroblast cultures, consisting in morphological alterations in the tendon matrix associated with increased active MMP2, and defective cell polarization in vitro. 

By immunofluorescence we found that collagen VI organization was altered in tendon cultures from both BM and UCMD patients. In fact, in patients’ cells collagen VI formed large aggregates, scattered in the ECM, and apparently was not associated with the cell surface. Rotary shadowing analysis confirmed the immunofluorescence pattern, revealing that collagen VI aggregates consisted of tangled microfilaments scarcely connected with the cell processes. The presence of aggregates is widely reported in the ECM of skin fibroblasts and skeletal muscle of UCMD patients [29,30]. Similar alterations were also reported in the matrix of a UCMD patient carrying a homozygous mutation in *COL6A2* [16]. The formation of protein aggregates is consistent with the effect of UCMD mutations on collagen VI assembly, which may affect both intracellular and extracellular steps. However, the presence of collagen VI aggregates in the BM tendon fibroblast culture was an unexpected finding, since the analysis of a muscle culture from the same patient showed a normal organization of collagen VI (patient BM2 in [29]). On the basis of previous studies performed on skin and skeletal muscle cultures, BM and UCMD mutations were reported to have a different impact on collagen VI expression pattern; in fact, while UCMD mutations cause collagen VI absence/reduced expression and aggregates, BM mutations induce subtle changes of protein organization, mainly detectable by rotary shadowing and electron microscopy analysis [31,32]. Thus, the severity of collagen VI defects reported here in BM tendon fibroblast cultures may suggest that collagen VI mutations have a differential impact on collagen VI organization depending on the tissue; although, we cannot exclude the involvement of additional regulatory mechanisms.

We previously found that the transmembrane proteoglycan NG2, encoded by the *CSPG4* gene plays a regulatory role in collagen VI organization within the pericellular matrix of normal tendon [16]. In normal proliferating tendon fibroblasts collagen VI showed an early association with the cell membrane in areas expressing NG2 proteoglycan. In UCMD cells, we found a clear reduction of NG2 by Western blot analysis which correlated with a dramatic reduction of the co-localization rate with collagen VI, as indicated by the *k2* coefficient. The distribution of NG2 was also altered in the BM culture, although the protein level was not apparently affected, as indicated by Western blot analysis. RT-PCR analysis of *CSPG4* did not reveal any defects at the transcriptional level in both BM and UCMD cells, suggesting that NG2 defects may be related to post-transcriptional events. The expression of NG2 was reported to be altered in the muscle of UCMD patients and in a *Col6a1*-/- murine model [27]. We recently found that NG2 was also reduced in tendon cultures of a UCMD patient [16]. These data indicate that changes in collagen VI expression also cause a parallel change in NG2 expression. It is possible that membrane-associated collagen VI might protect NG2 from proteolytic degradation. 

We previously reported that the binding of collagen VI to NG2 is essential for the orientation of tendon fibroblasts migration in vitro. We found that collagen VI-NG2 co-localized at the trailing edge of migrating cells, providing an anchorage to the substrate [16]. In order to define the impact of collagen VI mutations on cell migration, we subjected BM and UCMD cultures to a scratch wound assay in vitro. It is interesting to note that collagen VI was almost absent at the trailing edge of migrating cells and correlated with a reduced expression of NG2 proteoglycan. In addition, the number of incorrectly oriented cells was markedly increased in UCMD cultures, and, to a lesser extent, in BM cells. Tracking analysis of migrating cells showed that during migration UCMD and BM cells displayed a random trajectory. These data further support our hypothesis that alterations of collagen VI-NG2 axis affect cell orientation during migration.

A large number of regulatory molecules interact with collagen VI, including metalloproteinase MMP2 [33]. Interestingly, we found an increase of MMP2 in conditioned medium from both BM and UCMD tendon fibroblasts. RT-PCR analysis of *MMP2* mRNA did not show obvious differences with respect to normal cells, pointing to the involvement of post-transcriptional regulatory mechanisms in MMP2 activation. It is interesting to note that α2 chain of collagen VI modulates the activity of MMP2 by sequestering pro-MMP2 in the extracellular matrix, and blocking proteolytic activity [33]. It is conceivable that mutated collagen VI loses this specific function, resulting in the increase of active MMP2. In agreement, a moderate increase in MMP2 was observed in *Col6a1*-/- mice, a collagen VI null model [27], and in the tendon culture of a patient with a *COL6A2* mutation [16]. 

Although the active MMP2 protein was similarly increased in BM and UCMD conditioned media, the gelatinolytic activity was significantly increased only in the UCMD culture, pointing to a differential regulation of protein activity in UCMD with respect to BM. The regulation of MMP2 activity involves several mechanisms, such as transcription, regulation of mRNA half-life, secretion, intra- or extracellular localization, enzyme activation, and inhibition by specific and nonspecific cellular protease inhibitors [34]; thus, determining the actors responsible for such a differential regulation in BM and UCMD requires further extensive studies. 

MMP2 is involved in the initiation and progression of fibril growth and matrix assembly during tendon development [35]. Consistent with the role of MMP2 in collagen fibril organization, we found that the expression of collagen type I and XII were affected in areas of collagen VI accumulation in BM and UCMD tendon fibroblast cultures. Also fibronectin, a pericellular matrix component of tendon cells, displayed changes in its three-dimensional arrangement, similar to what we previously described in collagen VI-deficient fibroblasts [36]. The presence of anomalous aggregates in the matrix may also contribute to the ECM alterations. It was proposed that mutated collagen VI accumulates in the matrix affecting correct binding with extracellular matrix partners.

We also detected ECM alterations in the tendon biopsy of both BM and UCMD patients. Ultrastructural analysis revealed alterations of fibril morphology and a significant reduction in the number of large fibrils. These data correlate with fibril abnormalities reported in the skin of UCMD patients [37], in the tendons of collagen VI myopathy mouse models [27,28,38], and in the tendon of a UCMD patient [16].

Altogether, our data indicate that COL6 mutations affect both the in vivo and in vitro organization of the matrix tendon, resulting in defects consistent with dysfunctional fibrillogenesis. Fibril alterations were reported in some forms of Ehlers-Danlos syndrome (EDS) with hypermobile phenotype [39] and in an animal model of EDS with joint phenotype [40], suggesting the involvement of common pathophysiological pathways in this group of connective tissue disorders. Fibril abnormalities have also been considered a consequence of decreased loading [41], disuse, and aging-related sarcopenia [42]. Our data, however, point toward a primary tendon dysfunction in BM and UCMD, as indicated by the presence of alterations in tendon derived cells, demonstrating a causative effect of collagen VI mutation in determining the tendon phenotype. 

Contractures are common to several forms of muscular dystrophies and age-related sarcopenia and represent a highly debilitating condition which worsens the motor capability and quality of life of affected people. Joint contractures are currently treated with surgery with scant success. Understanding the biologic mechanism of contractures is an area explored very little and by few research teams. The occurrence of contractures in diseases with different genetic origins points to a common pathogenic feature. However, the causative mechanisms of contractures are still unclear.

The contractures hitherto considered only as a consequence to the fibro-adipose replacement of the skeletal muscle, with loss of elasticity and elongation, greatly limit the motor function in patients with myopathies. Our report on tendon alterations in collagen VI-related myopathies sheds light on collagen VI-related defects involved in contracture development. Future therapeutic strategies could take advantage of restoring the proper relationship between ECM components and improving tendon cell migration.

## Supplementary Materials

The following are available online at https://www.mdpi.com/2073-4409/9/2/409/s1, Figure S1: Biochemical, colocalization and ultrastructural analysis of patients’ cultures and tissue.
