# Supplementary material for: Tendon Extracellular Matrix Remodeling and Defective Cell Polarization in the Presence of Collagen VI Mutations
Source: Cells. 2020 Feb 11;9(2):409. doi: 10.3390/cells9020409 (PMC7072441; doi:10.3390/cells9020409)
Supplement: Supplementary file 1 [file cells-09-00409-s001.pdf]

Supplementary materials

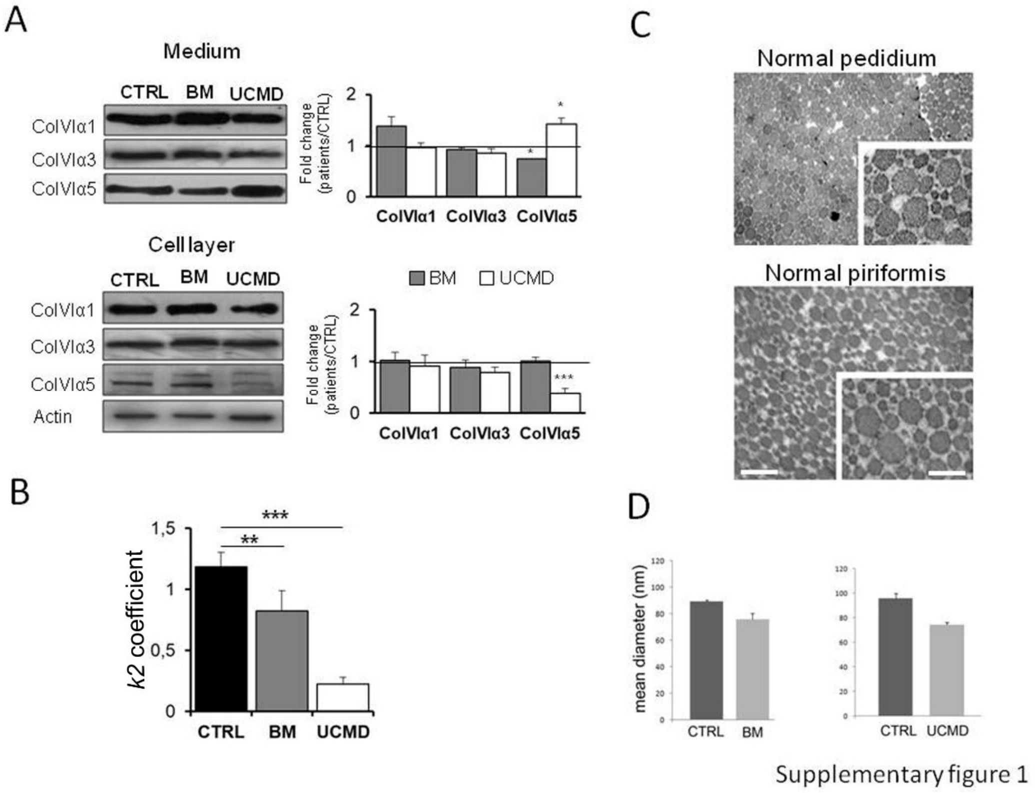

Figure S1. Biochemical, colocalization and ultrastructural analysis of patients' cultures and tissue. **(A)** Western blot analysis of COL6 α1,α3 and α5 chains in control (CTRL), BM and UCMD conditioned medium and cell lysates. Densitometric analysis of COL6 chains shows a moderate reduction of the 5 chain in cell layer of the UCMD patient. The respective protein levels in BM and UCMD patients' cells and media were compared to the control (showed by the dark line, set as 1). Error bars indicate SE. (**B**) Colocalization analysis of samples double labelled with anti-COL6 and NG2 proteoglycan antibodies. The graph shows *k2* coefficient. (**C**) Representative images of transversal sections of normal pedidium and piriformis tendons showing collagen fibrils with regular profiles. Scale bar, 150 nm. (**D**) Graph shows mean diameter (nm) of collagen fibrils of pedidium of UCMD patient and piriformis of BM patient, compared with the respective control.
